# Effects of Different Doses of Multienzyme Supplementation on Growth Performance, Duodenal pH and Morphology, and Carcass Traits in Broilers Fed Diets with an Increasing Reduction in Energy

**DOI:** 10.3390/ani13142378

**Published:** 2023-07-21

**Authors:** Mosaad Hashim, David Gonzalez-Sanchez, Alexandra Wealleans, Mohamed Abdelkader, Salah Abdel Rahman El-Safty, Abdel Rahman Y. Abdelhady

**Affiliations:** 1 Applied Feed Research House (AFRH), Orabi Community, Obour City 11828, Egypt; mosaadhashim@gmail.com (M.H.); salahsafty2004@yahoo.com (S.A.R.E.-S.); abdelrahman_abdelhady@agr.asu.edu.eg (A.R.Y.A.); 2 Kemin Animal Nutrition and Health, 2200 Herentals, Belgium; alexandra.wealleans@kemin.com (A.W.); mohamed.abdel-kader@kemin.com (M.A.); 3 Department of Poultry Production, Faculty of Agriculture, Ain Shams University, Hadayek Shoubra, Cairo 11241, Egypt

**Keywords:** multienzyme, NSP, broilers, carbohydrase, protease, feed cost, intestinal morphology, carcass traits

## Abstract

**Simple Summary:**

Feed cost accounts for around 70% of the total production cost in broiler production, and finding strategies to reduce it is of paramount importance to achieving sustainable profitability. Among other strategies to reduce feed cost, the use of carbohydrases and proteases has been widely implemented in many poultry operations around the globe. In broiler production, the use of these enzymes can improve performance and can be supplemented to reduce energy–cost, with the aim of improving overall profitability. Moreover, these enzymes can also provide benefits to some carcass traits. The present study aims to evaluate the effects of supplementing different doses of a multienzyme complex containing carbohydrases and a protease on growth performance, duodenal pH and morphology, and carcass traits in broilers fed diets that are increasingly reduced in energy. Our results showed that this multienzyme complex could improve performance and duodenal morphology and reduce abdominal fat in broiler chickens fed reduced-energy diets.

**Abstract:**

This study evaluated the effects of supplementing different doses of a multienzyme (KZP) consisting of carbohydrases and a protease on growth performance, duodenal pH and morphology, and carcass traits in broilers fed diets with increasing reductions in energy. One thousand two hundred one-day-old broiler chicks were allocated to five dietary treatments with eight replicates of 30 birds each: a positive control diet formulated to meet Arbor Acres’ nutritional requirements (PC); a negative control diet reformulated to 80 kcal/kg less than the apparent metabolizable energy (AME) of the PC (NC1); a negative control diet reformulated to 120 kcal/kg less than the AME of the PC (NC2); an NC1 diet supplemented with 300 g/t of KZP (NC1 + KZP300); and an NC2 supplemented with 500 g/t of KZP (NC2 + KZP500). Growth performance was measured throughout the study. At 35 days, 10 birds per treatment were randomly selected and euthanized for a carcass trait evaluation, and samples of the duodenum were collected for morphological examination and pH level determination. The final average body weight and feed conversion ratio were better (*p* < 0.05) for the broilers in the NC1 + KZP300 group compared to those in NC1, NC2 and NC2 + KZP500 groups and were similar to those of the PC birds (*p* > 0.05). Birds from the NC1 + KZP500 group showed a better (*p* < 0.05) final body weight and feed efficiency compared to the NC1 and NC2 groups. The villus height was greater (*p* < 0.05) for the PC and NC1 + KZP300 groups compared to the rest of the treatments. The crypt depth was longer (*p* < 0.05) for the NC1 and NC2 groups compared to the NC1 + KZP300 group. The supplementation of KZP to both the NC1 and NC2 diets reduced (*p* < 0.05) the abdominal fat %. This study demonstrates that supplementing energy-reduced diets with KZP improved performance in broiler chickens.

## 1. Introduction

Non-starch polysaccharides (NSPs) are the major components of dietary fiber and comprise cellulose, non-cellulosic polymers and pectic polysaccharides [1]. Viscous grains, such as wheat and barley, are rich in water-soluble NSP, which can increase the viscosity of the digesta and reduce nutrient digestion and absorption [2]. Increased viscosity can decrease passage rate, reduce feed intake, and increase microbial proliferation in the intestine [3,4]. As a result, growth performance and profitability are impaired [5,6,7]. On the other hand, grains like corn and sorghum are low in viscous NSP and do not create problems of increased viscosity. However, insoluble NSP may act as a physical barrier to enzymes, thus hindering efficient starch and protein digestion [8]. Supplementing poultry diets with NSP-degrading enzymes can decrease intestinal viscosity and the cell-wall encapsulating effect, leading to improved nutrient digestibility and growth performance [9].

The pancreatic secretion of amylase during the first days after hatching is limited and may impair starch digestibility and early growth [10,11]. On the other hand, the very high feed intake of broilers from modern genetics may limit starch digestibility [12]. Therefore, the exogenous supplementation of amylase can complement the endogenous supply, allowing for more complete starch degradation and matching the requirement for improved early growth performance [13]. 

The digestibility of crude protein (CP) and amino acids (AAs) in poultry diets can be impaired by the presence of anti-nutritional factors (ANFs) in soybean meal [14,15]. The values of the protein digestibility of feed ingredients reported in the literature suggest that there are substantial amounts of non-digested protein passing through the gastrointestinal tract (GIT) [16]. Considering the consistent increase in the cost of protein ingredients, this non-digested protein fraction may represent significant economic losses. Moreover, non-digested protein fractions from feed materials can reach the hindgut, serving as substrates for pathogen proliferation, such as *Clostridium perfringens* [17]. Exogenous protease supplementation haven shown to improve the CP and AA digestibility of broiler diets [18], allowing for reductions in the cost of feed [19,20], environmental impact [21] and alterations in the broilers’ intestinal ecosystems [22,23,24].

The supplementation of broiler diets with combinations of xylanase, amylase and protease has been extensively researched [25,26,27,28,29,30,31,32,33,34,35,36]. It has been shown to improve nutrient digestibility [26,28,29,31,33,34,35,36] and growth performance [26,28,29,35]. However, beneficial effects on performance in diets based exclusively on corn and soybean appear to be less consistent [25,27,30] or are dose dependent [28]. Supplementing corn–soy diets with more diversified NSP-degrading enzyme activities has shown to improve broiler performance [37,38,39,40], although this approach can lack consistency [41,42,43]. 

Dietary energy is one of the most expensive nutrients, alongside AA, in broiler diets [44]. Supplementing corn–soy diets with combinations of exogenous enzymes may allow for the reformulation of diets to lower the energy–cost expense [26,29,43], thus reducing the feed cost per kg of BWG and increasing the income over feed cost of a broiler operation. However, there is scarcity of research on the effects of supplementing broiler diets with more diversified NSP-degrading enzyme activities combined with amylase and protease on performance, carcass traits and intestinal morphology. This study aimed to evaluate the dietary supplementation of a multienzyme consisting of xylanase, β-glucanase, cellulase, amylase and protease on the growth performance, duodenal pH and morphology and carcass traits in broilers fed diets with increasing reductions in energy. 

## 2. Materials and Methods

### 2.1. Broilers, Diets, and Housing

A 35-day trial was conducted at the Broiler Research Unit of AFRH (Applied Feed Research House Evaluation & Innovation), Egypt. All experimental procedures were in line with commercial practices and were approved by the experimental animal care and research ethics committee of the Aims Shams University agriculture sector committee (approval No. 5-2023-1). 

A total of 1200 one-day-old Arbor Acres female broiler chicks (45.13 ± 0.91 g at hatch) were sourced from a commercial hatchery. The birds were randomly allocated to five dietary treatments with 240 birds each: a positive control diet formulated to meet Arbor Acres’ nutritional requirements (PC); a negative control diet reformulated to 80 kcal/kg less than the apparent metabolizable energy (AME) of the PC (NC1); a negative control diet reformulated to 120 kcal/kg less than the AME of the PC (NC2); an NC1 diet supplemented with 300 g/t of KZP (NC1 + KZP300); and an NC2 supplemented with 500 g/t of KZP (NC2 + KZP500). The multienzyme complex used in this study was KEMZYME Plus dry (Kemin Europa NV, Herentals, Belgium), which is based on Endo-1,4-beta-xylanase 35,000 U/g, Endo-1,3(4)-beta-glucanase 2350 U/g, Endo-1,4-beta-glucanase 18,000 U/g, Alpha-amylase 400 U/g and Bacillolysin 1700 U/g.

The experiment was carried out in 40 floor pens (replicates) of 2 m^2^ (15 chicks/m^2^ stocking density). Each group contained 8 replicates of 30 broilers housed together as an experimental unit. The replicates were homogenized to provide similar (*p* > 0.1) body weights (BWs) at the start for the different groups. During the experiment, all broilers were reared on sawdust-bedding litter. Each day, the building’s temperature and ventilation were monitored and adjusted in accordance with breed guidelines. Natural light and fluorescent bulbs placed above the pens supplied a consistent lighting program (0–3 days, 24 h/light; 4–7 days, 23 h/light; and 8-final age, 20 h/light). 

A corn-and-soybean diet was provided to the birds. The feeding program consisted of three phases: a starting diet from days 0 to 14, a grower diet from days 14 to 28, and a finisher diet from days 28 to 35. The starter diet was fed as crumbles, and the grower and finisher diets were fed as pellets. The pelleting conditions (<60 °C) were acceptable for the heat-stability of all the enzymes. Feed and water were provided ad libitum throughout the study. The ingredient composition and nutritional profiles of the diets are shown in Table 1.

### 2.2. Growth Performance Evaluation

All birds were weighted individually after their arrival from the hatchery and on days 14, 28 and 35. To provide pen-level data, individual body weights were averaged. To determine the feed intake (FI) and feed conversion ratio (FCR), feed bags and all remaining feed in the feeders were weighed. The daily mortality per pen was recorded. The birds were slaughtered on day 35.

### 2.3. Carcass Traits 

A total of 10 birds with an average live BW of 2000 g were selected and slaughtered for the evaluation of carcass traits. This selection protocol was conducted to avoid the creation of potential differences in carcass yield by differences in the final live BW. After slaughtering and bleeding the chickens, their feathers were removed, the carcasses were carefully eviscerated, and each carcass was weighed. The entire breast with wings and the thighs, drumsticks, and abdominal fat were removed from the carcass and weighed individually. The liver, gizzard, heart, spleen, and bursa were also weighed individually. The carcass traits were expressed as a percentage of the slaughtering weight.

### 2.4. Duodenal pH and Morphology

On day 35, 10 birds per treatment were randomly selected and euthanized, and duodenal samples from the pylorus to the distal portion of the duodenal loop were collected for morphological examination and pH level determination. Before emptying the digesta, the pH of the duodenum was measured in triplicate samples using a digital pH meter (AD1030 ADWA Instruments Inc., Szeged, Hungary). The average value obtained from three measurements per bird was used for statistical analysis. The collected samples of the duodenum were then emptied of digesta and fixed in 10% formal saline, washed, dehydrated, cleared, and embedded in paraffin. As described by Bancroft and Stevens, the paraffin-embedded blocks were sectioned at thicknesses of 4–5 μm, and stained with hematoxylin and eosin. Computer-generated images of the samples were examined for morphometric changes in the villi height and crypt depth, using a digital camera (AmScope 14 MP) with an associated image analysis software (SKU: MU1403, AmScope, Irvine, CA, USA) attached to a digital microscope (Leica 2135, Germany). Two measurements of villus height (VH) and crypt depth (CD) were made from each slide, and the average values of these measurements were used for statistical analysis. The ratio of villus height/crypt depth (VH:CD) for each replicate was calculated from the average measurement.

### 2.5. Statistical Analysis

Data are presented as means with overall standard errors of the mean (SEMs) and were analyzed in the Fit Model platform of JMP 15 (SAS Institute, Cary, NC, USA) with the experimental diet as the main factor. The pen was considered the experimental unit for performance, carcass traits, and duodenal pH and morphology. No outlier data were identified or excluded from the dataset. The means were compared using Tukey’s test. In all statistical analyses, differences were considered significant at *p* < 0.05; *p* < 0.1 was considered to indicate a near-significant trend.

## 3. Results

### 3.1. Growth Performance

Table 2 presents the performance results per feeding period and overall (0 to 35 days). Mortality was considered low (<5%) and did not differ among the different treatments. By day 28, the broilers in the NC1 + KZP300 group were heavier (*p* < 0.05) compared to those in the NC1, NC2, and NC2 + KZP500 treatment groups, while no difference (*p* > 0.05) was found when they were compared to the birds in the PC group. Between days 14 and 28, broilers from the PC, NC1 + KZP300, and NC2 + KZP500 groups were heavier (*p* < 0.05) and showed a better (*p* < 0.05) FCR compared to broilers from the NC1 and NC2 groups. Between days 28 and 35, broilers from the NC2 + KZP500 group showed a higher FI (*p* < 0.05) compared to the birds in the NC1 + KZP300 and PC groups, while no difference (*p* > 0.05) was found compared to the NC1 and NC2 groups. The average BWG of broilers from the NC1 + KZP300 group was even significantly higher compared to the NC1 and NC2 groups (*p* < 0.05), though not different (*p* > 0.05) from the PC and NC2 + KZP500 groups. The FCRs were better for the broilers in the NC1 + KZP300 (*p* < 0.05) and PC groups compared to broilers in the NC1, NC2 and NC2 + KZP500 groups. The FCRs for the broilers from the NC1 and NC2 + KZP500 groups were better (*p* < 0.05) compared to NC2. At day 35, the final BW was higher (*p* < 0.05) for the broilers in the NC1 + KZP300 group compared to those in the NC1, NC2, and NC2 + KZP500 groups and not different (*p* > 0.05) compared to that of the PC birds. The FI was not different (*p* > 0.05) among treatments. The FCR was significantly reduced in broilers of the PC and NC1 + KZP300 (*p* < 0.05) groups compared to all other treatments and significantly better for the NC2 + KZP500 group compared to the NC1 and NC2 groups (*p* < 0.05). Broilers from the NC2 group had the worst FCR (*p* < 0.05) compared to all other treatments.

### 3.2. Carcass Traits 

Table 3 presents the carcass trait results. An evaluation of the selected carcass traits showed that the thigh %’s of the NC1 + KZP300 and NC2 + KZP500 birds were higher (*p* < 0.05) compared to those of the NC1 and NC2 broilers and not different (*p* > 0.05) from the PC broilers. The supplementation of the KZP in both the NC1 and NC2 diets reduced (*p* < 0.05) the abdominal fat %. The birds from the NC + KZP500 treatment showed a lower (*p* < 0.05) abdominal fat% compared to all other treatments. No differences (*p* > 0.05) were detected for the rest of the evaluated carcass traits. 

### 3.3. Duodenal pH and Morphology

The impact on morphometric changes in the villi and pH of the duodena of the birds at 35 days, according to treatment, are shown in Table 4. The VH was longer (*p* < 0.05) for the PC and NC1 + KZP300 groups compared to the rest of the treatments. The CD was higher (*p* < 0.05) for the NC1 and NC2 groups compared to the NC1 + KZP300 group. The VH:CD was higher (*p* < 0.05) for the NC1 + KZP300 group compared to the NC2 + KZP500, NC2, and NC1 groups and no different compared to the PC. Group The pH was higher (*p* < 0.05) for the NC2 group compared to all other treatments. 

## 4. Discussion

### 4.1. Growth Performance

Feed accounts for 60–70% of the total cost of broiler production [45]. Modern broilers’ genetic potential for growth performance is largely driven by a high supply of both energy and AA [46]. Achieving this potential implies formulating diets at a high cost in an increasingly volatile environment [47]. Therefore, keeping the balance between broiler growth performance and production profitability becomes a major challenge. 

Modern strains of chickens are unable to accurately adjust their feed intake in response to varying dietary energy levels [48]. Similarly, in the present study, FI was not impacted by either ME reduction or KZP supplementation. However, the BW and FCR were negatively affected by reducing ME levels by 80 and 120 kcal/kg. Similarly, Haetinger et al. [49] showed that reducing the ME by 60 and 100 kcal/kg broiler diets had no influence on the FI but impaired both the BWG and FCR from 1 to 42 days. These findings suggest that the broilers did not respond to the lower ME with a higher feed intake and could not overcome the reductions in the ME to maximize the BWG and FCR. The multienzyme combination used in the present study, when supplemented at 300 mg/kg to diets reduced by 80 kcal/kg ME, restored the cumulative FCR and BWG to the level of the birds on the PC diet. Other studies also reported this compensatory effect of ME without performance impairment following multienzyme supplementation [26,29,50,51,52]. However, from 0 to 14 days, neither FI, BWG, nor FCR were affected by the ME level or KZP supplementation. Another previous research study [51] also did not find any effect of the ME level or KZP supplementation in the first two weeks of the growing period. It could be hypothesized that greater variability among pen-replicates during early rearing compared to subsequent growing phases could have influenced the detection of a statistical differences. 

### 4.2. Carcass Traits

The effect of supplementing enzyme combinations on the carcass traits of broiler chickens has shown to be inconsistent [43,53,54,55]. Similarly, in our research work, the supplementation of the multi-enzyme did not result in a better dressing percentage or breast meat yield. However, the diets that were reduced in ME and supplemented with the multi-enzyme decreased abdominal fat deposition. Interestingly, an extensive review by Fouad et al. [56] did not describe the use of enzymes among feed additives influencing fat deposition. The strongest ME reduction without enzyme supplementation decreased abdominal fat deposition compared to the higher- and intermediate-energy diets. This is in agreement with the research of Fouad et al. [56], who described energy as one of the most important factors to modify to reduce body fat deposition in poultry. Several previous studies reported reduced abdominal fat deposition and no impact on dressing percentage or other important carcass traits following dietary energy reductions [57,58,59,60] in broiler chickens. The effect of multi-enzyme supplementation on abdominal fat deposition requires further research and validation. 

### 4.3. Duodenal pH and Morphology

The villus height to crypt depth ratio is regarded as a one of the most relevant indicators for intestinal health [61]. However, the underlying mechanisms of the effect of supplementing diets based on corn and soybean meal with enzymes on intestinal morphology are not fully understood. In the present study, a greater reduction in ME (−120 kcal/kg) decreased the VH:CD ratio and increased the pH, and KZP supplementation restored these values to the same levels found in the birds fed the more energetic positive control diet. Similar findings were reported by Alqhatani et al. [52], with multienzyme supplementation improving VH. However, in contrast with our study, the energy level did not influence intestinal morphology. This is in agreement with Karimi et al. [62], who found that the VH of the duodenum was increased via the dietary supplementation of either β-betaglucanase or β-mannanase but was not influenced by the energy level of the diet. However, Zou et al. [63] did not find any effect of energy level nor enzyme supplementation on the VH:CD ratio in the duodenum at 42 d of age. Literature showing changes in duodenal pH following multienzyme supplementation alongside a reduction in ME is scarce. In the present study, the supplementation of 500 mg/kg of KZP to the broiler diet with an ME alteration of −120 kcal/kg decreased the duodenal pH. However, Mathlouthi et al. [64] and Basmacioğlu Malayoğlu et al. [65] did not report differences in the pH of the intestinal digesta following the dietary supplementation of enzyme complexes based on xylanase and β-betaglucanase to wheat–barley- and wheat-based diets, respectively, in broiler chickens. It could be hypothesized that the improvement of growth performance reported in our study could be attributed to improved intestinal health due to a higher VH:CD ratio. However, this hypothesis requires further validation through an evaluation of the morphology in other intestinal segments as well as nutrient digestibility and a microbiome assessment.

## 5. Conclusions

The findings of the present study demonstrate that adding a multienzyme consisting of xylanase, β-glucanase, cellulase, amylase and protease to corn- and SBM-based diets with reductions in ME improved the BWG and FCR and duodenal morphology and reduced abdominal fat deposition. Both dietary reductions of ME (−80 and −120 kcal/kg) impaired the BWG and FCR. The higher dietary reduction of ME (−120 kcal/kg) reduced the VH:CD ratio, increased the pH in the duodenum, and reduced abdominal fat deposition compared to a more energetic positive control diet. The intermediate dietary reduction of ME (−80 kcal/kg) supplemented with 300 mg/kg of the KZP showed no difference in the BWG, FCR, VH:CD ratio, and duodenal pH and a reduction in abdominal fat deposition compared to the more energetic positive control diet. This could translate into substantial economic benefits for industrial broiler production.

## Figures and Tables

**Table 1:** Ingredients and nutrient compositions of the experimental diets.

	0–14 Days (Starter)			14–28 Days (Grower)			28–35 Days (Finisher)		
Ingredients (g/kg)	PC	NC1 ^1^	NC2 ^1^	PC	NC1 ^1^	NC2 ^1^	PC	NC1 ^1^	NC2 ^1^
Corn	584.00	586.02	562.52	649.21	636.29	621.10	701.83	680.95	669.78
Soybean meal, 46% CP	343.13	361.92	356.64	251.92	310.39	306.66	179.74	254.08	254.92
Soybean oil	10.00	2.00	2.00	10.00	6.92	5.99	15.00	15.00	13.20
Corn gluten meal, 60% CP	23.72	10.00	10.00	50.33	10.00	10.00	63.37	12.64	10.00
Limestone	11.65	12.81	12.93	11.39	11.10	11.18	12.07	11.70	11.75
Wheat bran	-	-	29.37	-	-	20.00	-	-	15.00
Monocalcium phosphate	11.09	11.00	10.63	10.31	10.07	9.82	9.92	9.62	9.41
Sodium chloride	1.76	1.92	1.85	1.45	1.95	1.90	1.14	1.77	1.77
Sodium bicarbonate	2.43	2.57	2.24	2.86	2.16	2.19	3.32	2.42	2.40
L-Lysine HCl	3.89	3.39	3.44	4.83	3.30	3.33	5.82	3.87	3.80
DL-Methionine	3.57	3.67	3.66	2.94	3.25	3.24	2.86	3.25	3.27
L-Threonine	1.16	1.10	1.12	1.16	0.97	0.99	1.33	1.10	1.10
Vitamin-mineral premix ^2^	3.00	3.00	3.00	3.00	3.00	3.00	3.00	3.00	3.00
Betaine	0.25	0.25	0.25	0.25	0.25	0.25	0.25	0.25	0.25
Phytase ^3^	0.10	0.10	0.10	0.10	0.10	0.10	0.10	0.10	0.10
Bio-emulsifier ^4^	0.25	0.25	0.25	0.25	0.25	0.25	0.25	0.25	0.25
Feed cost (EUR/t) ^5^	425.8	416.7	414.3	414.7	404.5	402.2	407.0	397.1	394.0
Cost difference vs. PC (EUR/t)		−9.1	−11.5		−10.2	−12.5		−9.9	−13.0
Calculated nutrient composition (%, as fed basis)									
Dry matter	87.68	87.56	87.56	87.68	87.56	87.54	87.72	87.62	87.59
ME, kcal/kg	2900	2820	2780	3000	2920	2880	3100	3020	2980
Crude protein	22.65	22.65	22.65	20.5	20.5	20.5	18.5	18.5	18.5
Crude fat	3.70	2.92	2.92	3.84	3.50	3.42	4.43	4.38	4.21
Crude fiber	3.08	3.17	3.36	2.73	2.99	3.12	2.46	2.77	2.89
SID Lysine	1.32	1.32	1.32	1.20	1.20	1.20	1.10	1.10	1.10
SID Methionine	0.68	0.68	0.68	0.60	0.60	0.60	0.58	0.58	0.58
SID Methionine + cysteine	0.96	0.96	0.96	0.87	0.87	0.87	0.82	0.82	0.82
SID Threonine	0.81	0.81	0.81	0.73	0.73	0.73	0.67	0.67	0.67
SID Arginine	1.32	1.33	1.33	1.10	1.20	1.20	0.91	1.05	1.05
SID Tryptophan	0.22	0.23	0.23	0.19	0.20	0.20	0.15	0.17	0.17
Ca	0.95	0.95	0.95	0.85	0.85	0.85	0.85	0.85	0.85
Digestible phosphorous	0.48	0.48	0.48	0.45	0.45	0.45	0.43	0.43	0.43
Na	0.15	0.15	0.15	0.14	0.14	0.14	0.14	0.14	0.14
Cl	0.22	0.22	0.22	0.22	0.22	0.22	0.22	0.22	0.22

^1^ To create the experimental treatments, a multienzyme consisting of xylanase, β-glucanase, cellulase, amylase and protease was added at the expense of corn as follows: NC1 + KZP300, 300 mg/kg (Endo-1,4-beta-xylanase 10,500 U/kg, Endo-1,3(4)-beta-glucanase 705 U/kg, Endo-1,4-beta-glucanase 5400 U/kg, Alpha-amylase 120 U/kg, and Bacillolysin 510 U/kg; NC2 + KZP500, 500 mg/kg (Endo-1,4-beta-xylanase 17,500 U/kg, Endo-1,3(4)-beta-glucanase 1175 U/kg, Endo-1,4-beta-glucanase 9000 U/kg, Alpha-amylase 200 U/kg and Bacillolysin 850 U/k, where 1 U of endo-1,3(4)-beta-glucanase is the amount of enzyme that liberates 0.0056 micromoles of reducing sugars (glucose equivalents) from barley beta-glucan per minute at pH 7.5 and 30 °C; 1 U of endo-1,4-beta-glucanase is the amount of enzyme that liberates 0.0056 micromoles of reducing sugars (glucose equivalents) from carboxymethyl-cellulose per minute at pH 4.8 and 50 °C; 1 U of alpha-amylase is the amount of enzyme that liberates 1 micromole of glucose from a cross-linked starch polymer per minute at pH 7.5 and 37 °C; 1 U of endo- 1,4-beta-xylanase is the amount of enzyme that liberates 0.0067 micromoles of reducing sugars (xylose equivalents) from birchwood xylan per minute at pH 5.3 and 50 °C; and 1 U bacillolysin unit (U) is the amount of enzyme that solubilizes one microgram of azo-casein substrate per minute at pH 7.5 and 37 °C. ^2^ Provided per-kilogram diet: retinyl acetate, 3.50 mg; cholecalciferol, 0.1 mg; α-tocopherol acetate, 25 mg; menadione, 3 mg; thiamine, 2.0 mg; riboflavin, 7 mg; pyridoxine, 4.0 mg; cobalamin, 0.020 mg; niacin, 50 mg; calcium pantothenate: 15 mg; Cu (from copper sulphate), 9.0 mg; Fe (from ferrous sulphate), 35 mg; I (from potassium iodate): 1 mg; Mn (from manganese sulphate), 85 mg; Se (from sodium selenite), 0.35 mg; Zn (from zinc oxide), 80 mg. ^3^ 6-Phytase 500 FTU/kg. Axtra^®^ PHY. Danisco Animal Nutrition, IFF. ^4^ LYSOFORTE^®^ EXTEND, a proprietary combination of lysolecithin, synthetic emulsifier and monoglycerides manufactured by Kemin Europa NV, Herentals, Belgium. ^5^ Corn: 0.285 EUR/kg; soybean meal, 46% CP: 0.550 EUR/kg; soybean oil: 1 EUR/kg; corn gluten meal, 60% CP: 0.850 EUR/kg; L-Lysine HCl: 1.2 EUR/kg; DL-Methionine: 4 EUR/kg; L-Threonine: 1.6 EUR/kg; wheat bran: 0.260 EUR/kg.

**Table 2:** Effects of the dietary supplementation of KZP on the growth performance of broilers in each experimental group, measured at different growth stages.

	PC	NC1	NC2	NC1 + KZP300	NC2 + KZP500	SEM	*p*-Value
BW d0, kg	0.046	0.045	0.045	0.045	0.045	0.0003	0.7743
BW d14, kg	0.550	0.536	0.531	0.536	0.523	0.0068	0.0963
BW d28, kg	1.565 ^ab^	1.438 ^c^	1.416 ^c^	1.581 ^a^	1.535 ^b^	0.0110	<0.001
BW d35, kg	2.077 ^ab^	1.921 ^c^	1.872 ^d^	2.098 ^a^	2.047 ^b^	0.0111	<0.001
BWG 0–14, kg	0.505	0.491	0.486	0.491	0.478	0.0069	0.1121
BWG 14–28, kg	1.015 ^a^	0.902 ^b^	0.885 ^b^	1.046 ^a^	1.013 ^a^	0.0089	<0.001
BWG 28–35, kg	0.511 ^ab^	0.483 ^bc^	0.456 ^c^	0.516 ^a^	0.512 ^ab^	0.0073	<0.001
BWG 0–35, kg	2.031 ^ab^	1.876 ^c^	1.827 ^d^	2.053 ^a^	2.002 ^b^	0.0112	<0.001
FI 0–14, kg	0.595	0.589	0.593	0.584	0.574	0.0072	0.2896
FI 14–28, kg	1.521	1.511	1.488	1.543	1.505	0.0141	0.1046
FI 28–35, kg	0.836 ^b^	0.855 ^ab^	0.867 ^ab^	0.832 ^b^	0.890 ^a^	0.0123	0.0134
FI 0–35, kg	2.952	2.955	2.948	2.960	2.970	0.0185	0.9284
FCR 0–14	1.180	1.200	1.220	1.190	1.204	0.0100	0.0917
FCR 14–28	1.500 ^b^	1.676 ^a^	1.682 ^a^	1.478 ^b^	1.487 ^b^	0.0090	<0.001
FCR 28–35	1.640 ^c^	1.768 ^b^	1.900 ^a^	1.613 ^c^	1.742 ^b^	0.0136	<0.001
FCR 0–35	1.421 ^d^	1.538 ^b^	1.574 ^a^	1.412 ^d^	1.452 ^c^	0.0048	<0.001

PC: positive control diet formulated to meet Arbor Acres nutrition requirements; NC1: negative control diet reformulated to 80 kcal/kg lower than the AME of the PC; NC2: negative control diet reformulated to 120 kcal/kg lower than the AME of the PC; NC1 + KZP300: NC1 supplemented with 300 mg/kg of KZP; NC2 + KZP500: NC2 supplemented with 500 mg/kg of KZP. SEM: standard error of the mean (overall), *n* = 8 replicates per treatment (30 birds per replicate). BW: body weight; BWG: body weight gain; FI: feed intake; FCR: feed conversion ratio. a–d Values with different superscripts in the same row were significantly different (*p* < 0.05).

**Table 3:** Effect of the dietary supplementation of KZP on the selected carcass traits and yields (expressed as percent of slaughtering weight) of broilers in each experimental group.

	PC	NC1	NC2	NC1 + KZP300	NC2 + KZP500	SEM	*p*-Value
Dressing ^1^, %	74.56	74.01	72.14	75.29	75.20	0.1984	0.513
Breast ^2^, %	32.22	31.75	30.02	31.55	31.14	0.1300	0.241
Thigh ^3^, %	16.61 ^ab^	16.00 ^b^	15.95 ^b^	17.03 ^a^	16.70 ^a^	0.0937	0.010
Drumstick ^3^, %	8.67	8.15	8.07	8.90	8.77	0.0958	0.425
Abdominal fat, %	1.33 ^a^	1.29 ^a^	1.17 ^b^	1.18 ^b^	1.08 ^c^	0.0496	0.040
Liver, %	2.32	2.38	2.33	2.52	2.50	0.0660	0.244
Gizzard, %	0.96	1.09	0.89	0.90	1.00	0.0188	0.152
Heart, %	0.31	0.32	0.33	0.37	0.36	0.0039	0.363
Spleen, %	0.07	0.08	0.06	0.08	0.06	0.0023	0.203
Bursa, %	0.06	0.05	0.05	0.08	0.05	0.0025	0.452

PC: positive control diet formulated to meet Arbor Acres nutrition requirements; NC1: negative control diet reformulated to 80 kcal/kg lower than the AME of the PC; NC2: negative control diet reformulated to 120 kcal/*kg* lower than the AME of the PC; NC1 + KZP300: NC1 supplemented with 300 mg/kg of KZP; NC2 + KZP500: NC2 supplemented with 500 mg/kg of KZP. SEM: standard error of the mean (overall), *n* = 10 birds per treatment. ^1^ Eviscerated carcass (with neck and abdominal fat) ^2^ With wings, skin, and bones. ^3^ With skin and bones. a–c Values with different superscripts in the same row were significantly different (*p* < 0.05).

**Table 4:** Effect of the dietary supplementation of KZP on duodenal pH and morphology of broilers in each experimental group.

	PC	NC1	NC2	NC1 + KZP300	NC2 + KZP500	SEM	*p*-Value
Villus height, μm	1430.635 ^a^	1340.480 ^b^	1296.048 ^b^	1404.012 ^a^	1320.151 ^b^	14.327	<0.001
Crypt depth, μm	194.807 ^ab^	198.384 ^a^	199.936 ^a^	181.204 ^b^	186.504 ^ab^	3.848	0.0046
VH:CD ratio	7.362 ^ab^	6.785 ^bc^	6.509 ^c^	7.781 ^a^	7.098 ^bc^	0.158	<0.001
pH	5.502 ^b^	5.435 ^b^	5.884 ^a^	5.493 ^b^	5.469 ^b^	0.031	<0.001

PC: positive control diet formulated to meet Arbor Acres nutrition requirements; NC1: negative control diet reformulated to 80 kcal/kg lower than the AME of the PC; NC2: negative control diet reformulated to 120 kcal/kg lower than the AME of the PC; NC1 + KZP300: NC1 supplemented with 300 mg/kg of KZP; NC2 + KZP500: NC2 supplemented with 500 mg/kg of KZP. SEM: standard error of the mean (overall), *n* = 10 birds per treatment. a–c Values with different superscripts in the same row were significantly different (*p* < 0.05).

## Data Availability

The data presented in this study are available on request from the corresponding author.
